# An Optimized Metagenomic Approach for Virome Detection of Clinical Pharyngeal Samples With Respiratory Infection

**DOI:** 10.3389/fmicb.2020.01552

**Published:** 2020-07-10

**Authors:** Bo Liu, Nan Shao, Jing Wang, SiYu Zhou, HaoXiang Su, Jie Dong, LiLian Sun, Li Li, Ting Zhang, Fan Yang

**Affiliations:** ^1^National Health Commission of the People’s Republic of China Key Laboratory of Systems Biology of Pathogens, Institute of Pathogen Biology, Chinese Academy of Medical Sciences and Peking Union Medical College, Beijing, China; ^2^Division of Pulmonary and Critical Care Medicine, Beijing Chaoyang Hospital, Capital Medical University, Beijing, China

**Keywords:** respiratory virus, infection, viromes, COPD, metagenomics

## Abstract

Respiratory virus infections are one of the major causes of acute respiratory disease or exacerbation of chronic obstructive pulmonary disease (COPD). However, next-generation sequencing has not been used for routine viral detection in clinical respiratory samples owing to its sophisticated technology. Here, several pharyngeal samples with COPD were collected to enrich viral particles using an optimized method (M3), which involved M1 with centrifugation, filtration, and concentration, M2 (magnetic beads) combined with mixed nuclease digestion, and M4 with no pretreatment as a control. Metagenomic sequencing and bioinformatics analyses showed that the M3 method for viral enrichment was superior in both viral sequencing composition and viral taxa when compared to M1, M2, and M4. M3 acquired the most viral reads and more complete sequences within 15-h performance, indicating that it might be feasible for viral detection in multiple respiratory samples in clinical practice. Based on sequence similarity analysis, 12 human viruses, including nine *Anelloviruses* and three *coronaviruses*, were characterized. Coronavirus OC43 with the largest number of viral reads accounted for nearly complete (99.8%) genome sequences, indicating that it may be a major viral pathogen involved in exacerbation of COPD.

## Introduction

The ongoing outbreak of the novel coronavirus disease 2019 (COVID-19) caused by severe acute respiratory syndrome coronavirus 2 (SARS-CoV-2) poses a challenge for global public health^1^. SARS-CoV-2-infected patients experience severe pneumonia, pulmonary edema, SARS, multiple organ failure, and death (Ren et al., 2020). Individuals are usually most susceptible to respiratory disease caused by microbial infection in the winter (Romero-Espinoza et al., 2018). Chronic respiratory disease (CRS), such as chronic obstructive pulmonary disease (COPD), contributes to the major causes of morbidity and mortality in the elderly worldwide (Hewitt et al., 2016). It has been demonstrated that respiratory viruses, such as rhinovirus, can induce acute COPD exacerbation (Beckham et al., 2005; George et al., 2014). Multiplex-polymerase chain reaction (PCR) is a conventional assay used for virus detection that can provide some evidence of viral infection in COPD. However, it cannot provide virome characterization (Ko et al., 2019).

Currently, next-generation sequencing (NGS) is a relatively established technology in viral diagnostics (Parker and Chen, 2017), which includes investigation of viral infection and transmission events, identification of drug sensitivity or resistance, and new virus discovery (Grad et al., 2014; Greninger et al., 2017). Viral examination using a direct NGS strategy often has insufficient sensitivity owing to the low abundance of virus relative to the host (O’Flaherty et al., 2018). Usually, ultra-deep sequencing can increase the sensitivity of viral sequencing, especially when applied to the low copy numbers of viral samples (Yang et al., 2011). Metagenomic sequencing is one of the commonly used NGS strategies for viral genome sequencing (Houldcroft et al., 2017). The main challenge for metagenomic sequencing in viral genome sequencing is enriching small amounts of viral particles from large host and bacterial genomes (Yang et al., 2011; Houldcroft et al., 2017). Therefore, a series of methods have been applied for improving viral enrichment, such as DNase I treatment of the viral nucleic acids, removal of host rRNA, and filtration and centrifugation for sample pretreatment (Kleiner et al., 2015; Li et al., 2016; Goya et al., 2018). These methods are satisfactory for sequencing RNA virus genomes, although they would obviously not work with DNA viruses, which result in biased results for the whole viral genome distribution (Kleiner et al., 2015; Li et al., 2016; Goya et al., 2018). Ultracentrifugation or density gradient centrifugation have been applied to improve viral enrichment, although they are time-consuming and inconvenient for preparation of clinical samples for high-throughput sequencing (Wu et al., 2012; Parras-Molto et al., 2018). Studies have shown that samples have been subjected to one or two pretreatment procedures, including filtration, DNase and RNase enzyme digestion for host DNA, and RNA removal before DNA/RNA extraction from tested samples, for virome NGS sequencing (Wu et al., 2012; Parras-Molto et al., 2018). Meanwhile, some studies have described quantification of the effects for each pretreatment method in artificial samples (Hall et al., 2014; Lewandowska et al., 2017). When these pretreatment steps (filtration, nuclease) would be applied to clinical samples, they need to be reevaluated further. In addition, whether in-depth sequencing biases the composition of clinical respiratory virus samples is worthy of further investigation to improve virus detection sensitivity (Li et al., 2016).

Given the aforementioned technical uncertainties, the present study collected pharyngeal swabs from hospitalized patients with acute COPD exacerbation for NGS. The clinical samples were pretreated to enrich viruses using different combinations of methods, including centrifugation, polyvinylidene difluoride filtration (0.22-μm pore size) for removing eukaryotic cell- and bacterium-sized particles, 100-K centrifugal filtration for concentration, AMPure XP beads and RNA clean XP for removing host DNA and RNA, and then digesting samples in a cocktail of DNase and RNase enzymes for maximal virome retrieval. The following metagenomic sequencing methodology not only obtained genomic sequences of viruses from clinical samples, but four different treatments of each sample were evaluated in further detail. Based on NGS sequencing and comparisons of genome composition and sequence similarity, the genomes of 12 human viruses were characterized. This demonstrated the complete or partial genome sequences of 12 human viruses, including *Anelloviruses* and *coronaviruses*.

## Materials and Methods

### Ethics Statement

This project was approved by the Ethics Committee of Beijing Chaoyang Hospital in Capital Medical University and the Ethics Committee of the Institute of Pathogen Biology, Chinese Academy of Medical Sciences & Beijing Union Medical College. The informed written consent was acquired from all patients.

### Clinical Samples and RNA Extraction

The pharyngeal swabs from three hospitalized patients were immersed in a virus sampling tube containing 3 mL of maintenance medium (Yocon, China), vortexed for 30 s, and then immediately stored at −80°C. They were finally transported to the laboratory by dry ice shipment after 1 month, according to the requirements of biological safety. The patients, who were male, all had a history of COPD. Their average age was 55.5 years. They were hospitalized owing to acute lower respiratory tract infection in June 2019 at Beijing Chaoyang Hospital in Capital Medical University. Each sample was dispatched into three 1.5-mL Eppendorf tubes in 460-μL aliquots and selectively pretreated to enrich viral particles with three different methods, denoted with the codes M1–M3. In some cases (M1–M3), the samples were first centrifuged at 14,000 rpm for 10 min to remove cellular debris. The supernatants were separately filtered through a 0.22-μm membrane filter and 100-K centrifugal filters (Merck Millipore Ltd.) in order to exclude the remaining cellular debris and concentrate the samples in a volume of 100 μL. In other situations (M2–M3), the concentrated samples were then mixed with DNA clean XP and RNA clean XP (50 μL each of Agencourt AMPure XP beads and RNA clean XP, Beckman) for 5 min over ice to remove host RNA or DNA. The magnetic beads were subsequently removed using a magnetic separator. The supernatants of some samples (M3) were digested in a cocktail of DNase and RNase enzymes according to previously published methods (Wu et al., 2012) with a slight modification [10 U of Turbo DNase (Ambion), 10 U of RNase One (Promega), and 15 U of benzonase (Novagen) in 1 μL of DNase buffer (Ambion)] at 37°C for 30 min. Finally, the viral DNA and RNA of all samples were simultaneously extracted and eluted with 30 μL AVE buffer containing 1 μL RNase Inhibitor using a QIAamp viral RNA Minikit (Qiagen). For comparison, 140 μL of each original sample (M4) was indirectly isolated with the same kit without any of the above pretreatment.

### Retrotranscription and Double-Stranded cDNA Amplification of Virus

Sequence-Independent, Single-Primer-Amplification (SISPA) was performed according to the previously reported protocol (Chrzastek et al., 2017). Briefly, viral RNA was converted into first strand cDNA using K-8N primer in a total volume of 20 μL with SuperScript IV Reverse Transcriptase (SSIV) according to provided instructions (Thermo Fisher Scientific) (Chrzastek et al., 2017). Subsequently, 1 μL of Klenow Large Fragment (NEB) in NEB buffer was added to the retro-transcription reaction tube (final volume of 25 μL) to synthesize into double-stranded cDNA (ds-cDNA) according to provided manuals and guides (Chrzastek et al., 2017). The ds-cDNA amplification was performed with Premix Taq^TM^ (LA Taq^TM^ version 2.0 plus dye, Takala) in a final 50 μL reaction volume containing 4 μL of the ds-cDNA template and 1 μL of 20 pM primer K (5′-GACCATCTAGCGACCTCCAC-3′). The PCR amplification conditions were 94°C for 30 s, followed by 20 cycles of 94°C for 30 s, 50°C for 30 s, and 72°C for 2 min, with a final extension for 10 min at 72°C. Finally, the PCR product was purified with a 30 μL elution volume using the Min-Elute PCR extraction kit (Qiagen) and quantified through a Qubit 2.0 Fluorometer with a Qubit dsDNA HS Assay Kit (Thermo Fisher Scientific).

### Library Preparation and NGS Sequencing

Based on our optimized conditions, the ds-cDNA was diluted to a concentration of 200 pg/μL. An amount of 200 pg ds-cDNA (1 μL) was used to prepare a DNA sequence library and index incorporation with Nextera^®^ XT DNA Library Preparation kit (Illumina) and Nextera^®^ XT Index Kit (Illumina) according to the manufacturer’s instructions. AMPure XP beads were used as the clean-up reagent to optimize the library size at about 200 bp. Library fragments were verified using an Agilent 2100 analyzer (Agilent) following the manufacturer’s protocol. Sequencing was performed on a MiniSeq platform. A total of 12 libraries were equimolar pooled and sequenced in a single read of 150 bp in length with a 150 cycle MiniSeq Reagent High Output Kit (Illumina). The three control samples (M4) were then re-sequenced to about fivefold greater depth with an Illumina HiSeq PE150 (named M4-d).

### Read Mapping and Taxonomic Assignments

The raw sequencing reads were first trimmed for quality with Trimmomatic (V0.32) (Bolger et al., 2014) to obtain total reads and then screened as duplicate reads, low-complexity reads, and short reads (≤50 bp). After a series of empirical filters (data not shown in Table 1), surviving reads were mapped against the human reference genome (hg38) using mapping tool bowtie2 (Langmead et al., 2009). Apart from the host, the remaining reads were evaluated for origin by conducting alignments with the NCBI non-redundant nucleotide database (NT, downloaded in January 2019) with an e-value cutoff of 1 × 10^–10^ and maximal target sequencing of 5. To ensure the accuracy of classified information, the blast results were imported into MEGAN (Huson et al., 2016) (Metagenome Analyzer 6.15) using the MEGAN manual-recommended weighted lowest common ancestor (LCA) algorithm with parameters (e-value = 1 × 10^–10^; min Support = 1; weightedLCA = true; weighted LCA Percent = 75%) targeted to gain taxonomic information. Although the weighted LCA algorithm might assign reads more specifically than the naïve LCA algorithm as shown in the MEGAN manual, ambiguous taxonomy cannot be avoided entirely. Therefore, the study focused on virome analysis at taxonomic level (family), which covered 82–99% of the total viral reads, while taxonomic level genus and species covered 45–84% and 30–40%, respectively. To further investigate the origin of unknown reads, a bioinformatic tool SPAdes (V3.9.0) (Bankevich et al., 2012) was first employed to assemble the reads with no hits. Then, contigs with unassembled overlaps were merged using the SeqMan V7.1.0. The potential assembled contigs were then compared against the database of NR using Blastx with a looser e-value of 1 × 10^–5^ (Shi et al., 2016). Finally, unknown reads were remapped the assembled contigs to eliminate false negatives, the so-called “assembly-first strategy.”

**TABLE 1:** Summary of sequencing.

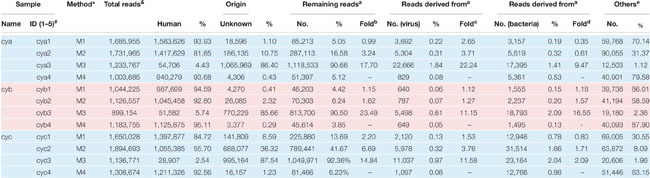

**FIGURE 1:**
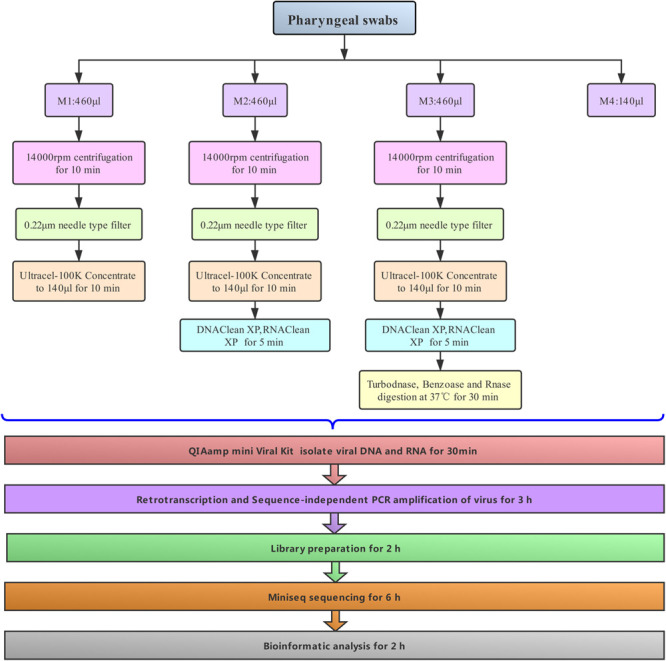
Schematic diagram for metagenomic virus sequencing.

*^#^Sequencing ID of sample. ^∗^Descriptions of M1, M2, M3, M4 methodologies are in section “Materials and Methods” and Figure 1. ^&^Total reads after quality control by Trimmomatic V0.32. ^*a*^Descriptions of remaining sequences are in section “Materials and Methods.” ^*b*^The fold values in the remaining sequences are relative to the M4 method for the same sample. ^*c*^The fold values in the virus sequences are relative to the M4 method for the same sample. ^*d*^The fold values in the bacterial sequences are relative to the M4 method for the same sample. ^*e*^Others contains reads of Archaea, reads of Eukaryota, reads with ambiguous taxa.*

### Assessment of Bias Degree Using “Combined Virome”

For a quantitative comparison of the capability for unbiased detection of viral pathogens among the four treatment methods, without any prior knowledge of the sample’s viral background, it is a priority to retrieve virome profiles comprehensively. Therefore, the four inferred taxonomic profiles, corresponding to each method, were combined into a single virome (called herein the “combined virome”) with the following screening criteria. A viral taxon must be supported by at least two unique reads, detected by either one method or different methods. Then, to assess the degree to which a method can identify most taxa of the combined virome in an unbiased manner, the taxa count obtained by each method was normalized to the taxa count of combined virome, the so-called “unbiased-index.”

Following this, we estimated the ability for unbiased detection against different viral properties (i.e., RNA virus, DNA virus, vertebrate-infecting viruses, enveloped virus, and non-enveloped virus) under these methods. The unbiased-index was adjusted to that of a specific viral property by normalizing the total number of viral taxa with such specific viral property against the total number of viral taxa of the combined virome with the same viral property.

### Nucleotide Sequence Accession Numbers

All 12 viral genome sequences were submitted to GenBank. The accession numbers for the three *coronaviruses* are MT501650 and MT501654–50165. The accession numbers for the nine *Anelloviridae* are MT501644–501649 and MT501651–501653. The GA II sequence data were deposited into the NCBI sequence reads archive under accession numbers PRJNA631867 (SAMN14917838–SAMN14917852).

## Results

### Clinical Sample Preparation and General Sequencing Results

In our workflow, metagenomics sequencing data were presented within less than 15 h (Figure 1). These generally stepwise methods included: 1. centrifugation + filtration + concentration (M1), 2. M1 + magnetic bead (M2), and 3. M2 + DNase and RNase enzymes (M3). Furthermore, each original sample without any pretreatment (M4) was used as an internal control to allow for evaluation of the effects of the enriched methods. An overview of the sequencing data obtained using is shown in Table 1. A total of 1.59 million valid sequence reads (2.38 Gb) were generated from the three samples with four different treatment methods, respectively, corresponding to the twelve subsamples (mean ± SD, 1,324,936 ± 329,307 reads). First, we evaluated the effect of different treatment methods (M1–M4) on the removal of human sequences, a major noise for viral enrichment. The highest percentage of human reads was obtained from the three control subsamples (cya4, cyb4, and cyc4, 93.78% on average) as well as subsamples treated with M1 (cya1, cyb1, and cyc1, 91.08% on average). In contrast, the lowest percentage of reads matched with human sequences (average: 4.23%) was achieved in the subsamples (cya3, cyb3, and cyc3) with M3 pretreatment. Moreover, the subsamples (cya2, cyb2, and cyc2) with M2 contained fewer human-related sequences, 86.46% on average. Based on these results, pretreatment with magnetic beads and DNase and RNase enzymes both led to a decrease in human-related sequences to different degrees.

To address the distribution of the remaining sequences excluded from the samples, while maintaining a low false-positive rate, the aligned sequences with a stringent cutoff (e-value up to 1 × 10^–10^) were converted into lists of viral taxa at different taxonomic resolutions. At the broadest taxonomic level (kingdom), the ratio of virus increased by an average of ∼15-fold for M3, ∼3-fold for M2, and ∼1.7-fold for M1 when compared to M4 (Table 1). The ratio of bacteria increased by an average of ∼7-fold for M3, ∼1.5-fold for M2, and ∼0.8-fold for M1 when compared to M4. By comparison, the bacterial ratios were only about half those of the viruses. In addition, we also observed that the proportion of reads of unknown origin was higher after the removal of host sequences by M3. Even though an assembly first strategy (see section “Materials and Methods”) and searches for homologs by loose cutoffs were carried out, an average of about 50% of reads in the M3 group still could not find their origin.

### Virome Constitutions of Each Sample

**FIGURE 2:**
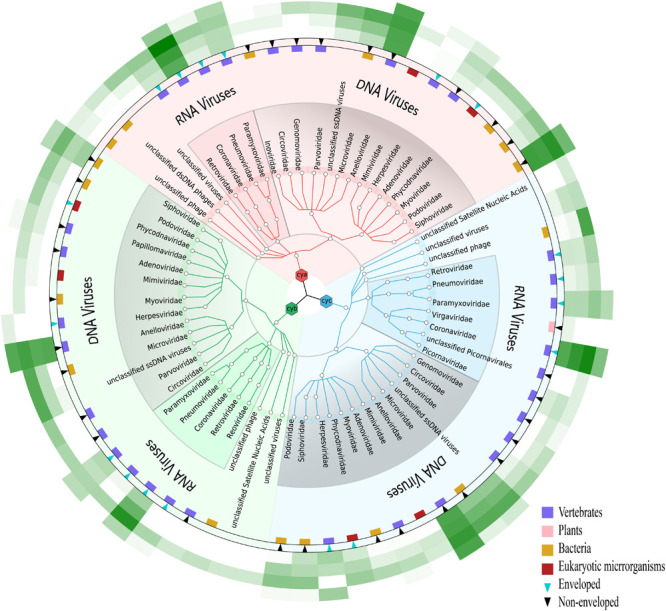
Viral community at the family level in three samples. Viruses for cya, cyb, and cyc are colored in pink, green, and blue, respectively. Presence of natural host and virus envelope is marked by a colored block and triangle, respectively. External green strips ranging from pretreatment method M1 (inner) to M4 (outer) represent viral read counts, which were normalized by valid sequencing reads in each subsample on a log scale.

Given that our study focused on the viral community, each viral taxon at the family level from the three samples was further categorized in the form of a tree structure (see Figure 2). The results noted with cya, cyb, and cyc showed that the total taxa numbered 21–23 conceivable viruses, including mostly mammalian viruses (families *Coronaviridae, Anelloviridae*, and *Paramyxoviridae*, labeled with pink blocks in Figure 2), phages of bacteria (order Caudovirales, families Microviridae and Siphoviridae, labeled with khaki blocks in Figure 2), and a small number of eukaryotic microorganisms (families Phycodnaviridae and *Mimiviridae*, labeled with red blocks). A heatmap of the four green strips in order, M1–M4 from inner to outside, showed that *Coronaviridae, Anelloviridae, and Siphoviridae* covered the main area with dark greens. We also observed that the M3 group showed the best enrichment with the exception of *Herpesviridae*, which were thought to have possibly integrated into the human genome (Tognarelli et al., 2019).

### Cross-Method Comparison of Viral Community

**FIGURE 3:**
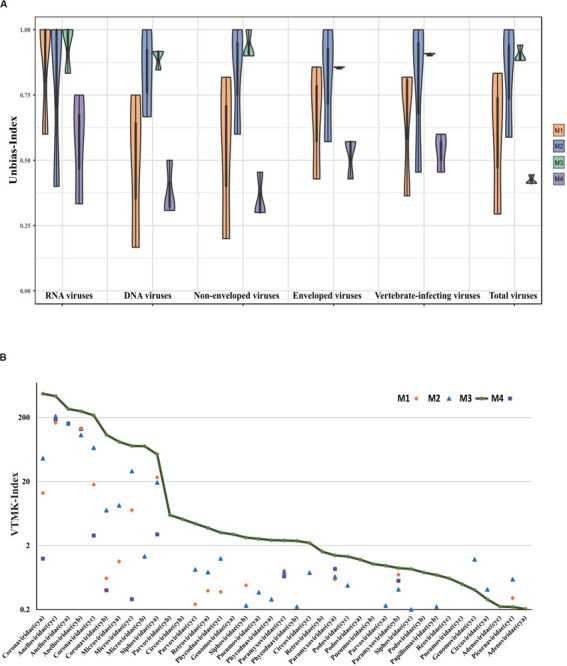
Comparative analysis of viral composition among different pretreatment methods. **(A)** Comparisons are shown for unbiased-index of six viral aspects based on three different samples, i.e., RNA virus, DNA virus, enveloped virus, non-enveloped virus, vertebrate-infecting viruses, and total number of viral families. **(B)** Viral reads were normalized by VTMK index, detected among three samples prepared by enrichment methods (M1, M2, and M3), and compared to non-enrichment method (M4). Viruses with VTMK index ≤0.2 are not shown in this figure.

It has been previously shown that detection of viral community composition using metagenomics sequencing may introduce various biases due to the intrinsic instability in different pretreatment methods as well as different viral properties (Kleiner et al., 2015). Thus, we performed a comparative analysis using an unbiased-index (see section “Materials and Methods”) rather than comparison of taxa counts between methods. Compared to M4 (see Figure 3A), the unbiased-index for M1, M2, and M3 improved at the family level, while M2 and M3 were enhanced significantly compared to M1. Both M2 and M3 showed no bias against specific viral properties (i.e., RNA virus, DNA virus, enveloped virus, vertebrate-infecting viruses, and non-enveloped virus).

For the quantitative evaluation of viruses detected in samples with different treatments, reads assigned to each viral taxa at family level were normalized by mean genome size corresponding to reference (see Supplementary Table S1) and total sequencing reads in each sample using the previously described VTMK index (number of valid tags per million sequences per kb of genome) (Yang et al., 2011). These viral families represented 82–99% of the total viral reads, and we therefore did not consider genus- or species-level assignments to be appropriate for quantitative analysis because the reads represent only 45–84% (genus) and 30–40% (species), respectively. Within most of the viral families, the VTMK indices were the highest for M3 samples, except for five non-enveloped viral families [Genomoviridae (cyc), Circoviridae (cya), Picornaviridae (cyc), Inoviridae (cya), and Virgaviridae (cyc)], which is probably due to their very low viral abundance in the sample (Figure 3B).

### Main Virus Verification of Viromes for Each Sample

**FIGURE 4:**
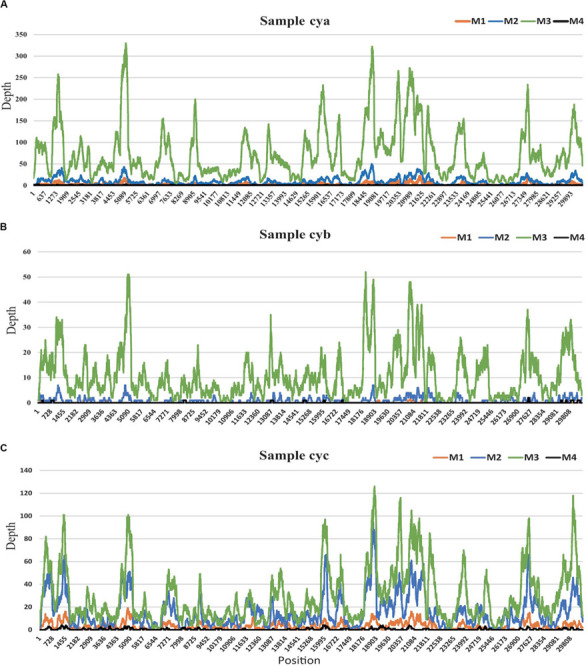
HCov-OC43 coverage map for three samples. **(A)** for sample cya, **(B)** for sample cyb, and **(C)** for sample cyc. In each sample, site depths based on read alignments on Hcov-OC43 genome are colored according to different experimental pretreatments: M1 (orange), M2 (blue), M3 (green), and M4 (black).

Among all 12 subsamples, the majority of the viral reads (average: ∼57%) aligned to a crucial respiratory virus (*Coronaviridae*, HCov-OC43) (Wylie, 2017; van Rijn et al., 2019). To explore the effects of the different methods on the efficiency of genome recovery, we compared the corresponding HCov-OC43 reference (MG197719) genome site sequencing depth profiles (see Figure 4). In those samples, the virus HCov-OC43 was steadily enriched by method M3, and an average of 99.8% (range, 97.8–99.9%) of the genome region was covered, whereas the coverage found in our sequence data in samples treated by the other methods was highly variable. The coverage of HCov-OC43 was 6–84% for M1, 50–98% for M2, and 4–32% for M4. We further verified a 440-bp region of the RNA-dependent reverse polymerase (RdRp) of OC43 viruses from the three original samples using RT-PCR with pan-coronavirus primers (Cor-F: GGTTGGGACTATCCTAAGTGTGA and Cor-R: CCATCATCAGATAGAATCATCATA) (Woo et al., 2005). Based on the same analysis, a total of 12 human viral species (see Supplementary Table S2 MT501644-501655) within the viromes were characterized, including *anelloviruses* (AV) and *coronaviruses*. This represented the complete consensus genome sequences of 12 human viruses (MT501644–MT501655).

### Impact of Sequencing Depth on Non-enriched Treatment

**FIGURE 5:**
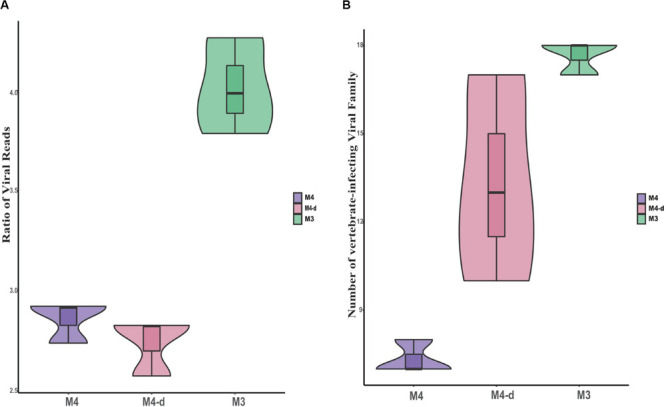
Comparison of viral reads and viral families at different sequencing depths. Comparisons are shown for the ratio of viral reads on a log scale **(A)** as well as absolute number of viral families **(B)** between non-enriched method M4, non-enriched method M4-d with fivefold higher sequencing depth, and enriched method M3.

To investigate the effect of sequencing depth on the ability to gain taxonomic information with non-enriched treatments, the three control samples (M4) were re-sequenced to about fivefold higher depth (see section “Materials and Methods”, M4-d), with an average read count of 6.6 million (mean ± SD, 6,610,925 ± 1,259,643 reads). Although the viral read count increased by about fourfold for M4-d when compared with M4, the relative ratio of viral reads did not show constant growth. We concluded that viral reads might not be proportionally enriched by the continuous increase in the depth of sequencing, as expected with unbiased sequencing. Moreover, we also observed trends in two aspects of the ratio of viral reads and the number of vertebrate-infecting viral families (see Figure 5). M4-d with five-fold increased depth did not show a more robust performance compared to M3. Together, the above data revealed that merely increasing the depth of sequencing could not make sufficient improvement for viral detection, but simply put more burdens on the follow-up analysis.

## Discussion

Common human respiratory tract viruses have been summarized for the period from 2009 to 2016, including *Rhinoviruses, Paramyxoviruses, Orthomyxoviruses, Coronaviruses, Adenoviruses, Parvoviruses, Herpesviruses, Anelloviruses, Papillomaviruses*, and *Polyomaviruses* (Wylie, 2017). To date, only one study characterized respiratory virome in the areas of COPD in 63 patients with acute COPD exacerbation from Norway, in which an obtained median of 11 million sequence reads per sample contained ∼93% of human reads, 3% of bacterial, 0.1% of viral, and 3% of unknown reads, as identified using NGS without any viral enrichment steps (van Rijn et al., 2019). In this study, we aimed to optimize the pretreatment method to enrich viral particles for NGS sequencing to investigate the virome in pharyngeal clinical samples from hospitalized patients with acute COPD exacerbation.

Currently, there are no standard methods for virome pretreatment for NGS sequencing. Target enrichment can enhance the sensitivity of respiratory virus genomic identification, but it is not aiming at the virome or unknown virus investigation (O’Flaherty et al., 2018). The present study found that the optimal workflow for metagenomics sequencing (Figure 1) could be completed in 15 h, which is time-saving compared to usual NGS sequencing methods (Thurber et al., 2009; Kohl et al., 2015). In order to optimize the methods of virus purification and enrichment, three pretreatments (M1, M2, and M3) were performed and M4 without any pretreatments was presented as the control in this work. The treatment supplied in M1 resulted in ∼1.7-fold increases in viral reads, with volumes increased threefold when compared to M4. M2, treated with AMpure DNA/RNA XP beads and used for removing the host genome, also gained ∼3-fold viral reads as compared to M4. Both M1 and M2 can improve the percentage of viral reads, although they had different effects. M3, a combination of a cocktail of DNase and RNase enzymes combined with M1 and M2, harvested the most viral reads (∼15-fold as compared to M4). Results presented in pharyngeal clinical samples showed that the greatest amounts of viral sequences from mammalian viruses were present in the families *Coronaviridae*, *Anelloviridae*, and *Paramyxoviridae*, *Coronaviridae* and *Anelloviridae* comprising the major part of the composition. Both *Coronaviridae* and *Anelloviridae* were detected in the same sample in the present research (Figure 4B), which was inconsistent with previous studies where *Anelloviridae* were not detected in the *Coronaviridae*-positive samples and *Coronaviridae* were not detected in the *Anelloviridae*-positive samples (van Rijn et al., 2019). We also observed that the M3 did not detecte *Herpesviridae*, which were thought to have possibly integrated into the human genome. Given that a cocktail of nucleases was used to digest nucleic acids from the host, the integrated *Herpesviridae* (Tognarelli et al., 2019) may not have been detectable in the M3 samples, but they were probably not the cause of COPD onset. The “cya” sample had more viral reads related to phages, including *Caudovirales* and families Siphoviridae and Podoviridae phages, which might derive from samples, ingredients, or oral bacteria. Interestingly, the three cases shared a similar virome and diverse viral reads percentage, perhaps due to the same period of hospitalization. Furthermore, M3 still acquired the most unknown reads (∼48%), although using a loose cutoff analysis. These might be intrinsic unknown nucleic acids in the sample that were proportionally amplified by NGS (Kleiner et al., 2015).

To assess whether the different pretreatment methods would bias the viral community composition and viral properties, we first proposed a concept named the unbiased-index to investigate the virome contents. Our results showed valid metagenomic data, including RNA virus, DNA virus, enveloped virus, vertebrate-infecting virus, and non-enveloped virus (Figure 3A). M2 and M3 methods applied to clinical samples maximized the viral composition and detection while minimizing bias. The M1 method involved in centrifugation, syringe-based filtration, and ultrafiltration was particularly effective for RNA examination, with no obvious effect on DNA compared to M2 and M3. Notably, the optimized methodology in M3 acquired a large number of viral reads and more viral taxa, which contributed to removing more host contamination by using a combination of centrifugation, filtration, Ampure Bead purification, and a cocktail of DNase and RNase enzymes. This finding indicated that the method was obviously superior compared to similar pretreatments (centrifugation, filtration, and nuclease treatment known as three-step treatment) that only targeted discovery of RNA viruses and did not include DNA virus (Hall et al., 2014). Agencourt AMPure XP has usually been used to purify DNA samples before sequencing in previous studies (Maricic et al., 2010). Here, it served as a pilot test to efficiently remove host nucleic acids without any bias with both magnetic AMPure XP beads and RNA clean XP, indicating its potential applications in the future. In our preliminary study, enzyme digestion with half of the amount of DNase and RNase enzyme for 30 min was superior to M2. However, it was inferior to M3 with Ampure Beads and an enzyme digestion treatment for sample pretreatment (data not shown). Furthermore, other pretreatments involving a combination of filtration, ultracentrifugation, and a cocktail of DNase and RNase enzymes have been successfully used in different studies with ultracentrifugation for 3 h and digestion at 37°C for 2 h (Donaldson et al., 2010; Wu et al., 2012). However, when compared to traditional enrichment of ultracentrifugation with low-throughput (six samples) and greater time taken (3 h) (Wu et al., 2012), the M3 assay allows simultaneous processing of 24–32 samples, depending on the centrifuge type, within 30 min.

The VTMK indexes for all viruses detected using different pretreatments (Figure 3B) elucidated that M3 subsamples had the highest values among all of the samples within most of the viral families, except for the five non-enveloped viral families [Genomoviridae (cyc), Circoviridae (cya), Picornaviridae (cyc), Inoviridae (cya), and *Virgaviridae* (cyc)], which was probably due to their very low viral abundance in the samples, creating unstable results. This indicates that the M3 method was optimal. Among the three samples, the majority of the viral reads aligned to a crucial respiratory virus (*Coronaviridae*, HCov-OC43), for which the VTMK index was calculated to be in the range of 0.5–850, indicating that samples with lower copy numbers of viruses can be enriched for detection. Moreover, the virus HCov-OC43 was reliably enriched by method M3, and an average of 99.8% (Figure 4, range, ∼97.8–99.9%) of the genome region was covered, whereas the coverage given by the other methods found in our sequence data was highly variable. The coverage of HCov-OC43 was 6–84% for M1, 50–98% for M2, and 4–32% for M4. Our results for M3 proved to be robust for viral verification but also to be accurate for viral genome analysis and evolutionary study. We further verified OC43 viruses in three original samples using RT-PCR with pan-coronavirus primers (Woo et al., 2005), indicating that it might have been one of the causes of acute COPD exacerbation (Wylie, 2017; van Rijn et al., 2019). Furthermore, the other nine *Anelloviridae* family viruses were also characterized (Supplementary Table S2). In sample “cyc”, Torque teno virus had 94% of reference identities (KJ082064), TT virus had 91% of AM712033 identities, and TT virus had 96% of AF345521 identities. Some new viruses, such as consensus nucleotide sequences that have only 48% of identities with Torque teno midi virus 8 (YP009505770), cover 75% of the genome (2,200 bp, data not shown). Those consensus sequences have been verified in the origin sample using PCR.

In our previous study, we used a metagenomic approach successfully to identify seven respiratory viruses in clinical samples without any enrichment, similar to M4 in the present study (Yang et al., 2011). The previous study showed that only about 0.05% of the valid sequence in each sample’s data with ultra-deep sequencing were available for further viral analysis, in comparison with an average of 91.6% of the host (human) genome and transcriptome, and 6.8% of unknown reads. Herein, we also investigated whether ultra-deep sequencing would have the ability to gain taxonomic information and increase the sensitivity of sequencing. When non-enriched treatment (M4) samples were re-sequenced to about fivefold higher depth (M4-d, Hiseq, Illumina), the viral read count for M4-d did not increase by about fivefold as expected, but only by fourfold. Moreover, M4-d with fivefold depth did not show stronger performance than M3. Therefore, the above data revealed that merely increasing the depth of sequencing could not provide sufficient improvement for viral detection, but increased the burden on the follow-up with bioinformatics analyses. Therefore, it seems cost-inefficient to obtain huge datasets for clinical usage.

The present study focused on a critical technology that enriches sample viral particles with a simple, feasible, optimized method (M3), resulting in a sequencing depth with a median of 1 M reads (150 M bp data per sample), which can provide an amount of viral reads that can cover the whole genome for analysis of viral polymorphism. The other key technology used was bioinformatics analysis using two concepts, the VTMK index and unbiased-index, to normalize the samples’ background information and viral constitution. Therefore, the M3 method can be used for viral detection in multiple clinical samples with higher sensitivity and high-throughput in a time-saving manner, especially when faced with a sudden outbreak of infectious diseases (e.g., COVID-19), using simplified bioinformatics analysis to accelerate the clinical application of NGS.

## Data Availability Statement

The datasets presented in this study can be found in online repositories. The names of the repository/repositories and accession number(s) can be found in the article/Supplementary Material.

## Ethics Statement

The studies involving human participants were reviewed and approved by the Ethics Committee of Beijing Chaoyang Hospital in Capital Medical University and the Ethics Committee of the Institute of Pathogen Biology, Chinese Academy of Medical Sciences & Beijing Union Medical College. The patients/participants provided their written informed consent to participate in this study.

## Author Contributions

TZ and FY designed the project. JW collected the samples. TZ, NS, HS, JD, LS, and LL conducted the experiments. BL, SZ, and TZ analyzed the data. TZ and BL wrote the manuscript. All authors contributed to the article and approved the submitted version.

## Conflict of Interest

The authors declare that the research was conducted in the absence of any commercial or financial relationships that could be construed as a potential conflict of interest.

## Footnotes

1 https://www.who.int/emergencies/diseases/novel-coronavirus-2019
