# Supplementary material for: An Optimized Metagenomic Approach for Virome Detection of Clinical Pharyngeal Samples With Respiratory Infection
Source: Front Microbiol. 2020 Jul 10;11:1552. doi: 10.3389/fmicb.2020.01552 (PMC7366072; doi:10.3389/fmicb.2020.01552)
Supplement: Supplementary file 1 [file Table_1.doc]

# Supplementary table1. Genome size of relevant viral families in this study

| **Viral family** | **Nature host*** | **Genome type*** | **Genome size (kb)*** | **Mean genome size (kb) ^#^** |
| --- | --- | --- | --- | --- |
| Anelloviridae | Vertebrates | DNA viruses | 3.8 | 3.8 |
| Adenoviridae | Vertebrates | DNA viruses | 35-36 | 35.5 |
| Circoviridae | Vertebrates | DNA viruses | 1.8-3.8 | 2.8 |
| Genomoviridae | Vertebrates | DNA viruses | 2.17 | 2.17 |
| Herpesviridae | Vertebrates | DNA viruses | 120-240 | 180 |
| Inoviridae | Bacteria | DNA viruses | 4.5-8 | 6.25 |
| Microviridae | Bacteria | DNA viruses | 4.4-6.1 | 5.25 |
| Mimiviridae | Eukaryotic microorganisms | DNA viruses | 1200 | 1200 |
| Myoviridae | Bacteria | DNA viruses | 33-244 | 138.5 |
| Papillomaviridae | Vertebrates | DNA viruses | 8 | 8 |
| Parvoviridae | Vertebrates | DNA viruses | 4-6 | 5 |
| Podoviridae | Bacteria | DNA viruses | 40-42 | 41 |
| Phycodnaviridae | Eukaryotic microorganisms | DNA viruses | 100-560 | 330 |
| Siphoviridae | Bacteria | DNA viruses | 50 | 50 |
| Coronaviridae | Vertebrates | RNA viruses | 27-32 | 29.5 |
| Paramyxoviridae | Vertebrates | RNA viruses | 15 | 15 |
| Picornaviridae | Vertebrates | RNA viruses | 7.1-8.9 | 8 |
| Pneumoviridae | Vertebrates | RNA viruses | 13-15 | 14 |
| Retroviridae | Vertebrates | RNA viruses | 7-11 | 9 |
| Reoviridae | Vertebrates | RNA viruses | 18.2-30.5 | 24.35 |

*Data are available from ViralZone <https://viralzone.expasy.org/> (Hulo C，et al. 2011. ViralZone: a knowledge resource to understand virus diversity. Nucleic Acids Res. 2011 Jan;39(Database issue):D576-82.)

^#^ Mean genome size = (minimal genome size +maximal genome size ) / 2
